# Cervical Pap screening among women living with HIV in Puerto Rico and the United States – Medical Monitoring Project, 2018–2021

**DOI:** 10.1016/j.gore.2024.101443

**Published:** 2024-06-26

**Authors:** Marievelisse Soto-Salgado, Lorena González-Sepúlveda, Maritza Cruz-Cortés, Michael I. Rivera-Morales, Sharee Umpierre, Jane R. Montealegre, Ana P. Ortiz

**Affiliations:** a University of Puerto Rico Comprehensive Cancer Center, Division of Cancer Control and Population Sciences, San Juan, PR, United States; b University of Puerto Rico Medical Sciences Campus, Graduate School of Public Health, Department of Biostatistics and Epidemiology, San Juan, PR, United States; c Puerto Rico Department of Health, Office of Epidemiology and Investigation, HIV/STD Surveillance Program, San Juan, PR, United States; d University of Puerto Rico Medical Sciences Campus, School of Medicine, Department of Obstetrics and Gynecology, San Juan, PR, United States; e University of Texas, MD Anderson Cancer Center, Division of Cancer Prevention and Population Sciences, Department of Behavioral Science, Houston, TX, United States

**Keywords:** Cervical cancer screening, Women, HIV, Medical Monitoring Project, Puerto Rico, United States

## Abstract

Our objective was to determine the prevalence of cervical Pap screening among women living with HIV (WLWH) in Puerto Rico (P.R.) and other selected United States (U.S.) jurisdictions. Additionally, we sought to compare selected characteristics of WLWH who underwent cervical Pap screening between P.R. and the other U.S. jurisdictions. We analyzed data from the 2018–2021 cycles of CDC’s Medical Monitoring Project (MMP), a national surveillance system among adults with HIV residing in P.R. (n = 218) and 22 other MMP jurisdictions (n = 3,653). Weighted percentages and 95 % confidence intervals (CIs) for selected characteristics were estimated. Prevalence ratios with predicted marginal means were calculated. An estimated 91.6 % and 84.6 % of WLWH underwent cervical Pap screening in P.R. and the other 22 MMP jurisdictions, respectively (Prevalence Ratio = 1.08, 95% CI = 1.03–1.13). Among WLWH who underwent cervical Pap screening, those in P.R. were more likely to be 50+ years of age, have a household annual income below $20,000, engage in binge drinking, never smoke, and have Medicaid/other public insurance than those in the other 22 MMP jurisdictions (p < 0.05). No differences were found between P.R. and the other 22 MMP jurisdictions in the percentage reporting higher than the median HIV-stigma score, experiencing HIV health care discrimination, and having ≥ 1 sexual partner in the past 12 months. Although cervical Pap screening rates among WLWH were higher in P.R. than in the other 22 MMP jurisdictions, both surpass the Healthy People 2030 target. Future research should assess adherence and compliance with updated cervical cancer screening guidelines.

## Highlights

• Puerto Rico and the United States show disparities in the uptake of cervical Pap screening among women living with HIV.
• Characteristics of women living with HIV undergoing cervical Pap screening differ in Puerto Rico and the United States.
• Variations in the utilization of cervical Pap screening were noted before and after the COVID-19 pandemic.

## 1 Introduction

Globally, it has been estimated that a total of 20 million women lived with HIV in 2022. (World Health Organization, 2022) The elevated number of women living with HIV (WLWH) is in part due to the widespread use of combination antiretroviral therapy (cART), which allows their expected lifespan to approach that of persons from the general population. (Thompson et al., 2021) This makes HIV care increasingly complex as WLWH can experience increased comorbidities across the lifespan, requiring furthered attention to issues associated with aging. For example, WLWH have an elevated risk of certain HPV-related cancers, notably cervical cancer. HIV can compromise cellular immunity, thereby increasing the susceptibility to persistent HPV infections, particularly those of oncogenic types. (Rahatgaonkar et al., 2021) Given that HPV is the primary cause of cervical cancers and precancerous cervical lesions, this co-infection poses a heightened risk for WLWH.

WLWH in the United States (U.S.) have a greater than four-fold increased risk of cervical cancer than women from the general population. (Abraham et al., 2013) Furthermore, findings from Ortiz and colleagues (Ortiz et al., 2014) suggests a higher excess incidence of cancer and HPV-related cancers among Hispanic PLWH in Puerto Rico (P.R.) than in racial/ethnic groups in the U.S. In P.R., a U.S. territory with a higher incidence of HIV/AIDS (Centers for Disease Control and Prevention, 2017), the adjusted standardized rates (ASR) for cervical cancer (ASR in PR: 9.1 per 100,000) among the general population was comparable to that of non-Hispanic black (NHB; ASR: 10 per 100,000), lower than U.S. Hispanics (USH; ASR: 12.7 per 100,000), and higher than non-Hispanic white (NHW; ASR: 5.9 per 100,000). (Ortiz et al., 2010) Hence, it suggests a higher prevalence of HPV infection or lower cervical cancer screening rates in USH, NHB, and P.R. (Ortiz et al., 2010) Moreover, the differences in incidence between these racial/ethnic groups could be related to disparities in cervical cancer screening uptake. (Ortiz et al., 2010).

Recent data has demonstrated that 83 % (81.7–84.3 %) of women aged 18 + years from the general population in P.R. were up to date with cervical cancer screening, comparable to the 84.5 % (84.3–84.8 %) in the U.S. (Gopalani et al., 2023) Women with certain sociodemographic characteristics, such as lower educational attainment, lower annual household income, without healthcare coverage, and smokers, were more likely to show a lower uptake of cervical cancer screening. (Gopalani et al., 2023) Meanwhile, the uptake of cervical cancer screening among WLWH in P.R. as compared to the U.S. has not been reported. This data is of utmost importance, considering that WLWH have aged and have tailored guidelines for cervical cancer screening. In 1995, new national recommendations were issued for WLWH, stating the need for more frequent Papanicolau (Pap) tests for cervical cancer screening. (Frazier et al., 2016) Guidelines have indicated that this population should undergo cervical cancer screening twice within the first year after their HIV diagnosis and annually thereafter, provided the results were normal during the first year. (Frazier et al., 2016) Recently, in July 2021, the World Health Organization (WHO) included 16 new and updated recommendations and good practice statements in its guidelines for screening and treatment to prevent cervical cancer in WLWH including HPV DNA detection in a screen, triage and treat approach starting at the age of 25 years and regular screening every 3 to 5 years. (World Health Organization, 2021).

Although cervical cancer screening should be part of the routine for clinical care among WLWH, there are many barriers to regular screening and appropriate follow-up and/or treatment for precancerous lesions and/or cervical cancer. (Kasraeian et al., 2020) A notable number of WLWH in the U.S. report not having had a Pap test within the previous 15 months (Barnes et al., 2018) (approximately 56 %). Therefore, our objective of estimating the prevalence of cervical Pap screening among WLWH in P.R. versus other U.S. jurisdictions could help measure the progress against the national cancer screening targets.

## 2 Methods

### 2.1 Study design and study population

We analyzed 2018–2021 data from the Medical Monitoring Project (MMP), a national surveillance system that reports representative estimates of characteristics and outcomes among adults diagnosed with HIV in the U.S. and Puerto Rico. Data were analyzed on WLWH residing in P.R. (N = 218) and the 22 other MMP U.S. jurisdictions (N = 3,653).

### 2.2 Data source

MMP uses a two-stage complex sample survey design. First, 16 states (including 6 separately funded jurisdictions) and P.R. were sampled from all U.S. states, the District of Columbia, and P.R. Next, simple random samples of adults with diagnosed HIV were drawn for each jurisdiction from the National HIV Surveillance System. MMP is conducted as a part of routine surveillance and is deemed non-research; data could be available from the corresponding author upon reasonable request. Response rates ranged from 40 %-45 % annually at the individual level, with a 100 % rate at the jurisdiction level. (Beer et al., 2019) This secondary data analysis was approved by the University of Puerto Rico Comprehensive Cancer Center IRB (#2022-09-84).

### 2.3 Measures

Data was collected through respondent interviews. The primary outcome was the self-reported cervical Pap screening in the past three years or since testing positive for HIV for those who received a diagnosis < 3 years ago. Covariates included demographic characteristics and social determinants of health (e.g., age, education, HIV stigma, and HIV health care discrimination), behaviors (e.g., number of sexual partners and binge drinking), and clinical characteristics (e.g., time since HIV diagnosis and current use of ART).

The median HIV stigma score, derived from a 10-item scale ranging from 0 (no stigma) to 100 (high stigma), assessed four dimensions: 1) personalized stigma during the past 12 months, 2) current disclosure concerns, 3) current negative self-image, and 4) current perceived public attitudes about people living with HIV. Meanwhile, the HIV health care discrimination experiences assessed seven forms of discrimination: 1) being treated with less courtesy than other people, 2) being treated with less respect than other people, 3) receiving poorer service than others, 4) having a doctor or nurse act as if he or she believed they were not smart, 5) having a doctor or nurse act as if he or she were afraid of them, 6) having a doctor or nurse act as if he or she were better than them, and 7) having a doctor or nurse not listen to what they were saying. These experiences were measured over the past 12 months. Participants were asked if they experienced these forms of discrimination never, rarely, some of the time, most of the time, or all the time. People were considered to have experienced health care discrimination if they answered 'rarely' or more often to any of the experiences.

### 2.4 Statistical analysis

Weighted percentages and accompanying 95% confidence intervals (CIs) were estimated for all characteristics. Prevalence ratios (PR) with predicted marginal means were calculated to compare percentages of WLWH who reported receiving a cervical Pap testing during the past 3 years, and the characteristics of WLWH who underwent cervical cancer screening in P.R. versus the 22 other U.S. MMP jurisdictions. All analyses were conducted using SAS survey procedures and SAS-callable SUDAAN.

## 3 Results

Table 1 shows the prevalence of WLWH, during 2018 to 2021, who have had a cervical Pap screening in P.R. and the 22 other U.S. MMP jurisdictions. The prevalence of cervical Pap screening was 8 % higher (PR: 1.08, 95% CI: 1.03–1.13) in P.R. than in the 22 other U.S. MMP jurisdictions. Annual percentages of WLWH who reported receiving cervical Pap testing during the period 2018–2021 ranged from 86.8 % to 97.9 % in P.R., whereas in the other 22 U.S. jurisdictions, it ranged from 83.2 % to 87.0 %. In 2020–2021, cervical cancer screening in P.R. was 10 % lower compared to 2018–2019 (PR: 0.90, 95% CI: 0.83–0.99; 87.0 % in 2020–2021 and 96.3 % in 2018–2019), while in the other 22 U.S. MMP jurisdictions (PR: 0.98, 95% CI: 0.94–1.02; 83.7 % in 2020–2021 and 85.5 % in 2018–2019) it did not vary significantly between periods.

**Table 1:** Prevalence of reported cervical Pap screening during the past three years among WLWH (aged 18 + years)—Medical Monitoring Project (MMP), United States, 2018–2021 cycles.

**Cervical pap test, last 3 years**	**P.R.**		**22 other MMP jurisdictions**^a^		**P.R. vs. 22 other MMP jurisdictions _(ref.)_**
	**N**	**Weighted** **Col % (95% CI)**	**N**	**Weighted** **Col % (95% CI)**	**PR** **(95% CI)**
Yes	199	91.6 (87.7–95.5)	3,106	84.6 (83.0–86.2)	1.08 (1.03–1.13)^b^
No	19	8.4 (4.5–12.3)	547	15.4 (13.8–17.0)	−

^Abbreviations:^ WLWH, women living with HIV; P.R., Puerto Rico; PR, prevalence ratio; CI, confidence intervals.

^Note:^ The primary outcome was the self-reported cervical Pap screening in the past three years or since testing positive for HIV for those who received a diagnosis < 3 years ago.

^a^ The following project areas were included: California, including Los Angeles County and San Francisco; Delaware; Florida; Georgia; Illinois, including Chicago; Indiana; Michigan; Mississippi; New Jersey; New York State, including New York City; North Carolina; Oregon; Pennsylvania, including Philadelphia; Texas, including Houston; Virginia; and Washington.

^b^ The results are statistically significant (p < 0.05).

Table 2 shows the characteristics of WLWH who reported undergoing cervical Pap screening in P.R. and the 22 other U.S. MMP jurisdictions. Among WLWH who underwent cervical Pap screening, those in P.R. were more likely than those in the 22 other U.S. MMP jurisdictions to be 50 + years of age (Table 2). WLWH in P.R. who received cervical Pap screening were around 39 % more likely to have a household annual income below $20,000 compared to WLWH in the other 22 MMP jurisdictions who received cervical Pap screening (PR: 1.39, 95% CI: 1.29–1.49). Of those who received cervical Pap screening, the prevalence of WLWH in P.R. who had Medicaid or other public insurance was 76 % higher than WLWH in the other 22 U.S. MMP jurisdictions (PR: 1.76, 95% CI: 1.56–2.00). Among WLWH who had cervical Pap screening, persons in P.R. were more likely to have an HIV diagnosis 10 + years ago (PR: 1.09, 95% CI: 1.01–1.17), engage in binge drinking (PR: 1.74, 95% CI: 1.18–2.57), and never smoked (PR: 1.22, 95% CI: 1.08–1.39) compared to those in the other 22 U.S. jurisdictions. The percentage of WLWH who reported higher than the median HIV stigma score, experiences with HIV health care discrimination, and ≥ 1 sexual partner in the past 12 months did not differ between P.R. and the other 22 MMP jurisdictions (Table 2).

**Table 2:** Prevalence of sociodemographic, health-related social determinants, clinical factors, and behaviors among WLWH (aged 18 + years) who reported undergoing cervical Pap screenings in the past three years— Medical Monitoring Project (MMP), United States, 2018–2021 cycles.

	**P.R.**		**22 other MMP jurisdictions**^a^		**P.R. vs. 22 other** **MMP jurisdictions**_(ref.)_
	n	Weighted Col % (95% CI)	n	Weighted Col % (95% CI)	PR (95% CI)
***Demographic characteristics and social determinants of health***					
**Age** (years)					
18–39	19	14.1 (7.4–20.9)	571	18.6 (16.8–20.5)	0.76 (0.46–1.24)
40–49	30	14.4 (9.1–19.7)	752	25.0 (22.9–27.1)	0.58 (0.39–0.84) ^b^
50–64	114	56.7 (48.8–64.6)	1500	47.0 (44.7–49.3)	1.21 (1.04–1.40) ^b^
≥ 65	36	14.8 (9.8–19.7)	283	9.4 (8.0–10.7)	1.58 (1.10–2.27) ^b^
**Education**					
< High school	49	21.7 (15.7–27.6)	808	26.4 (24.4–28.5)	0.82 (0.61–1.09)
High School diploma or GED	57	30.7 (23.4–38.1)	993	30.6 (28.5–32.7)	1.00 (0.78–1.29)
> High School	93	47.6 (39.7–55.5)	1304	42.9 (40.6–45.3)	1.11 (0.93–1.32)
**Annual Household Income**					
< $20,000	168	86.1 (80.9–91.4)	1728	62.0 (59.5–64.4)	1.39 (1.29–1.49) ^b^
≥ $20,000	28	13.9 (8.6–19.1)	1041	38.0 (35.6–40.5)	0.36 (0.25–0.54) ^b^
**Employment status**					
Currently employed	56	29.2 (21.9–36.5)	1173	36.8 (34.6–39.1)	0.79 (0.61–1.02) ^b^
Other status ^c^	142	70.8 (63.5–78.1)	1927	63.2 (60.9–65.4)	1.12 (1.01–1.25) ^b^
**Health care coverage or insurance for care or medications**					
Any private	39	19.4 (13.2–25.7)	830	27.0 (24.9–29.1)	0.72 (0.52–1.00) ^b^
Dual coverage Medicaid/Medicare or Medicare only	28	11.6 (7.1–16.1)	820	25.8 (23.8–27.9)	0.45 (0.30–0.66) ^b^
Medicaid or other public insurance	126	66.8 (59.6–74.0)	1192	37.9 (35.6–40.1)	1.76 (1.56–2.00) ^b^
Uninsured, including RWHAP only	−	−	238	9.3 (7.8–10.7)	0.23 (0.10–0.52) ^b^
**HIV stigma**^d^, median score (95% CI)	195	34.7 (30.8–38.6)	2923	34.1 (32.6–35.7)	−
**HIV stigma score ≤ 34.1 (median HIV stigma score among WLWH with cervical Pap screening status)**	85	43.0 (35.2–50.9)	1,358	46.9 (44.5–49.3)	0.92 (0.76–1.11)
**HIV stigma score > 34.1 (median HIV stigma score among WLWH with cervical Pap screening status)**	110	57.0 (49.1–64.8)	1,565	53.1 (50.7–55.5)	1.07 (0.93–1.24)
**Experienced any healthcare discrimination**^e^	43	21.8 (15.2–28.5)	594	20.7 (18.8–22.6)	1.06 (0.77–1.45)
***Clinical Characteristics***					
**Time since HIV diagnosis**					
< 5 years	15	7.2 (3.5–11.0)	326	9.7 (8.4–11.0)	0.74 (0.44–1.27)
5–9 years	21	9.6 (5.3–13.9)	434	13.7 (12.1–15.4)	0.70 (0.44–1.11)
≥ 10 years	163	83.2 (77.7–88.7)	2345	76.6 (74.6–78.6)	1.09 (1.01–1.17) ^b^
**Has a regular HIV care provider**	193	97.4 (95.2–99.7)	2969	94.9 (93.8–96.1)	1.03 (1.00–1.05)
**Current ART use**	198	99.7 (99.0–100.0)	2986	95.2 (94.0–96.4)	1.05 (1.03–1.06) ^b^
***Lifestyle and Sexual Behaviors***					
**Any alcohol use, past 12 months**	81	42.3 (34.4–50.1)	1568	51.7 (49.3–54.0)	0.82 (0.67–0.99) ^b^
**Binge drinking, past 30 days**	32	18.8 (12.0–25.6)	339	10.8 (9.3–12.2)	1.74 (1.18–2.57) ^b^
**Smoking status**					
Current	37	19.9 (13.1–26.8)	983	29.3 (27.1–31.4)	0.68 (0.48–0.97) ^b^
Former	31	15.1 (9.6–20.7)	556	17.7 (16.0–19.4)	0.85 (0.58–1.25)
Never	130	64.9 (57.2–72.7)	1561	53.0 (50.7–55.4)	1.22 (1.08–1.39) ^b^
**Sexual partners in the past 12 months**					
None	110	53.5 (45.6–61.4)	1535	49.2 (46.8–51.5)	1.09 (0.93–1.27)
At least one	89	46.5 (38.6–54.4)	1571	50.8 (48.5–53.2)	0.92 (0.77–1.09)

^Abbreviations:^ WLWH, women living with HIV; P.R., Puerto Rico; PR, prevalence ratios; CI, confidence intervals; GED, General Education Development (i.e., high school diploma equivalent); RWHAP, Ryan White HIV/AIDS Program; HIV, human immunodeficiency virus; ART, antiretroviral therapy.

^Note:^ The primary outcome was the self-reported cervical Pap screening in the past three years or since testing positive for HIV for those who received a diagnosis < 3 years ago. Estimates with a coefficient of variation ≥ 0.30 and estimates based on a denominator sample size < 30 were not reliable and therefore suppressed.

^a^ The following project areas were included: California, including Los Angeles County and San Francisco; Delaware; Florida; Georgia; Illinois, including Chicago; Indiana; Michigan; Mississippi; New Jersey; New York State, including New York City; North Carolina; Oregon; Pennsylvania, including Philadelphia; Texas, including Houston; Virginia; and Washington.

^b^ The results are statistically significant (p < 0.05).

^c^ It includes women who are homemakers, retired, students, unemployed, and unable to work.

^d^ ‘Median HIV stigma score’ was defined as the weighted median score on a 10-item scale ranging from 0 (no stigma) to 100 (high stigma) that measures 4 dimensions of HIV stigma: personalized stigma during the past 12 months, current disclosure concerns, current negative self-image, and current perceived public attitudes about people living with HIV, measured among persons aged ≥ 18 years with diagnosed HIV infection living in the U.S. and P.R.

^e^ HIV health care discrimination experiences were measured during the previous 12 months. The seven forms of HIV health care discrimination included being treated with less courtesy than other people, being treated with less respect than other people, receiving poorer service than others, having a doctor or nurse act as if he or she believed they were not smart, having a doctor or nurse act as if he or she were afraid of them, having a doctor or nurse act as if he or she were better than them, and having a doctor or nurse not listen to what they were saying. Participants were asked if they experienced this never, rarely, some of the time, most of the time, or all the time. People were considered to have experienced any health care discrimination if they answered “rarely” or more often to any of these 7 questions.

## 4 Discussion

Our study is the first to comprehensively examine cervical Pap screening prevalence among WLWH in P.R. and compare it to those in other selected U.S. jurisdictions. P.R. is one of the top ten U.S. states/jurisdictions with the highest number of cumulative AIDS cases and HIV prevalence. (Puerto Rico’s Department of Health, 2020) An assessment of the cervical Pap screening prevalence holds promise for guiding forthcoming public health initiatives for WLWH, potentially enhancing screening participation and mitigating disparities in cervical cancer-related morbidity and mortality. Our findings show that WLWH in P.R. and other U.S. MMP jurisdictions exceed the Healthy People 2030 target (79.2 %) for general population cervical cancer screening (U.S. Department of Health and Human Services, n.d.) by 16 % and 7 %, respectively.

In P.R., approximately 8 % more WLWH underwent cervical Pap screening compared to the 22 other U.S. MMP jurisdictions. Furthermore, a higher prevalence of cervical Pap screening was shown among WLWH when compared to the results obtained by Gopalani et al. among the general population. (Gopalani et al., 2023) These variations in cervical Pap screening may be attributed to differences in the cervical cancer screening guidelines, where WLWH are recommended to undergo screening more frequently and at a younger age than women in the general population. (World Health Organization, 2021) Therefore, our findings may suggest that WLWH adhere to the cervical cancer guidelines. Yet, in P.R., there was a 10 % reduction in report of cervical Pap screening from 2018–2019 to 2020–2021, possibly indicating reduced healthcare utilization during the COVID-19 pandemic or a shift in screening strategies that includes HPV testing, which extends the interval between screening tests. More research is needed to understand cervical screening uptake among WLWH, specifically if they are receiving appropriate screening (either Pap test only, or Pap testing and HPV co-testing) according to age.

Differences in sociodemographic, clinical, and lifestyle characteristics were observed among WLWH who received cervical Pap screening in P.R. compared to those who received cervical Pap screening in the other U.S. MMP jurisdictions. These differences between P.R. and the other U.S. MMP jurisdictions may reflect their respective demographic and sociocultural profiles, while differences in clinical attributes could also suggest disparities in healthcare access or variations during HIV follow-up care experienced by these women.

Our study is subject to some limitations. First, behavioral data results may be influenced by social desirability and recall bias due to their self-reported nature; however, nondifferential data misclassification in P.R. and the other U.S. MMP jurisdictions is expected. Second, participants' awareness of the specific type of cervical cancer screening received, whether cervical Pap test only, HPV test only, or HPV co-testing, may not have been certain, and could not be independently confirmed through medical record. Despite these limitations, this secondary data analysis has several strengths to be considered. First, the use of standardized data collection methods used across all MMP jurisdictions increases the internal validity of our study. Second, the selection of a representation sample of adults diagnosed with HIV in P.R. and the U.S., regardless of HIV care status, improves generalizability of our results. Despite the higher prevalence of cervical Pap screening observed for Puerto Rican WLWH, more research is necessary to assess adherence and compliance to updated cervical cancer screening guidelines. Also, research should assess the experiences and satisfaction with health services aimed at cancer prevention and care, including timeliness of follow-up for abnormal results and/or treatment for pre-cancerous lesions.

## CrediT authorship contribution statement

**Marievelisse Soto-Salgado:** Writing – original draft, Supervision, Methodology, Funding acquisition, Conceptualization. **Lorena González-Sepúlveda:** Writing – original draft, Visualization. **Maritza Cruz-Cortés:** Writing – review & editing. **Michael I. Rivera-Morales:** Writing – review & editing. **Sharee Umpierre:** Writing – review & editing. **Jane R. Montealegre:** Writing – review & editing. **Ana P. Ortiz:** Writing – review & editing.

## Declaration of competing interest

The authors declare the following financial interests/personal relationships which may be considered as potential competing interests: Dr. Ana P. Ortiz has previously served as a consultant for Merck outside the scope of this study. These financial associations did not influence the design, conduct, or interpretation of this study. No conflicts of interest were declared by the remaining authors.
